# Fusions4U: a resource of validated and annotated gene fusions in 328 cancer cell lines

**DOI:** 10.1186/s12885-025-15441-w

**Published:** 2025-12-20

**Authors:** Arianna Alamshahi, Helena Persson

**Affiliations:** Department of Clinical Sciences Lund, Oncology, Lund University Cancer Centre, Faculty of Medicine, Lund, Sweden

**Keywords:** Gene fusion, Fusion transcript, Cancer, Cell line, Kinase, MicroRNA host

## Abstract

**Background:**

Large-scale genomic rearrangements in cancer cells can lead to the creation of fusion genes which alter the expression or function of the partner genes. Some fusion genes act as tumour drivers and the use of kinase inhibitors in patients with specific fusions have led to breakthroughs in cancer therapy. The large clinical and scientific interest in fusions has led to the development of software that use RNA sequencing data to identify fusion transcripts. Unfortunately, fusion transcript callers output many predictions which lack underlying genomic rearrangements and large datasets with validated fusions are scarce.

**Results:**

This paper and the accompanying Fusions4U web application present a resource of validated and annotated gene fusions for 328 cell lines from the Cancer Cell Line Encyclopedia. Predicted fusion transcripts from Arriba and STAR-Fusion were analysed with our published validation pipeline that uses matched whole-genome sequencing data to identify discordantly mapped read pairs and candidate genomic breakpoints that support genuine fusion events. This resulted in 8,753 and 2,244 validated fusion transcripts for Arriba and STAR-Fusion predictions, respectively, with 1,596 fusions common to both. Additional layers of annotation include alternative splicing of fusion transcripts, kinases, microRNA host genes, genes in the COSMIC Cancer Gene Census, as well as known fusion gene pairs from the Mitelman Database of Chromosome Aberrations and Gene Fusions in Cancer and the TumorFusions dataset. We furthermore analysed information about fusion genes together with cell line data from the PRISM drug repurposing screening as an example of how this dataset can be used.

**Conclusions:**

This resource can be used to design experiments for functional studies and drug development, alone or in combination with publicly available information for the Cancer Cell Line Encyclopedia cell lines. The large collection of validated fusion transcripts with candidate genomic breakpoints can also be used in development and evaluation of bioinformatic tools for fusion transcript detection.

**Supplementary Information:**

The online version contains supplementary material available at 10.1186/s12885-025-15441-w.

## Background

In 1960, David Hungerford and Peter Nowell discovered the so-called Philadelphia chromosome while searching for chromosomal abnormalities in chronic myeloid leukaemia (CML) cells [1]. It was later determined that it arose from a translocation involving chromosomes 22 and 9, and that the breakpoints on each chromosome would lead to a fusion of the *BCR* and *ABL1* genes. In 1990, it was reported that the *BCR::ABL1* gene fusion results in an abnormal tyrosine kinase product. These findings have had cascading and lasting effects on both research and clinical practice in oncology and today tyrosine kinase inhibitors (TKIs) are the main course of treatment for patients with CML [1–3].

A gene fusion is the result of a DNA rearrangement that creates a chimera of two different genes. There are two main structural types of gene fusions: (1) the protein-coding sequences of two genes join to form a chimeric gene and (2) the promoter of one gene replaces the promoter of another gene and alters control of its expression, referred to as “promoter swapping” [4, 5]. These fusions can occur inter- or intra-chromosomally and arise from translocations, deletions, duplications, insertions, and inversions [6]. DNA-level gene fusions are then transcribed to fusion transcripts which have been found in many different cancer types, for example in the TumorFusions dataset [7] that is based on data from The Cancer Genome Atlas (TCGA) [8]. The fusion proteins which can form upon translation of fusion transcripts are a particular source of interest as they are potential drug therapy targets [2, 9] and can act as tumour drivers [10–12]. The large interest in gene fusions has led to the development of many different fusion transcript prediction software. These are based on detection of chimeric transcripts in RNA sequencing (RNA-Seq) data, which are more often available than whole genome sequencing (WGS) data, and ensure that the detected fusion events are expressed. For example, FusionGDB contains chimeric RNA predictions from several tools for a large number of samples, including tumours from the TCGA [13]. We and others have reported that fusion transcript predictions can have a high rate of false positives [14, 15]. The false positive rate is affected by many factors, such as RNA-Seq data quality (library preparation and sequencing) and the subsequent alignment [16], as well as the callers’ ability to differentiate chimeric fusion transcripts from other aberrant ones [14]. Unfortunately, large resources of validated fusions which can be used in methods development and tool comparison are scarce [14, 17, 18]. Synthetic spike-in RNAs and computationally simulated chimeric reads are therefore often used as substitutes, but these most likely do not recapitulate all the possible difficulties and error sources associated with real-world data.

Here, we have created a resource of validated gene fusions for 328 of the Cancer Cell Line Encyclopedia (CCLE) cell lines [19] using our recently developed pipeline for validation of predicted fusion transcripts with matched WGS data [17]. Fusion transcripts were predicted using both Arriba and STAR-Fusion with minimal downstream filtering before validation to avoid loss of genuine fusion events and increase the total number of reported gene fusions. The use of default settings and common transcript annotation will facilitate future use of our data. The addition of WGS data improves fusion transcript identification by detecting fusion events which can be found on both the RNA and DNA level, that is, both as a chimeric transcript in RNA-Seq data and as an underlying genomic rearrangement, often with a predicted breakpoint [17]. We have also annotated the gene fusions for instances of possible alternative splicing and identical predictions from both Arriba and STAR-Fusion; involvement of kinases [20] and microRNA (miRNA) host genes [21]; matches to the Mitelman Database of Chromosome Aberrations and Gene Fusions in Cancer [22] (hereafter referred to as the Mitelman database) and the TumorFusions dataset [7]; as well as for fusion partners implicated in the Catalogue of Somatic Mutations in Cancer Cancer Gene Census (COSMIC CGC) dataset [23]. Gene fusion datasets currently available for the CCLE cell lines have employed filtering [24] and a Bayesian classifier [25] to try to remove potential false positive fusion transcript predictions rather than performing validation with WGS data.

As we showcase here with an example using the Profiling Relative Inhibition Simultaneously in Mixtures (PRISM) drug screening dataset [24, 26], our data represent a useful resource for drug development and design of functional studies, especially when combined with the wealth of information that is available for the CCLE cell lines. Our resource of validated and annotated gene fusions can also be used in the development and evaluation of new bioinformatic tools for fusion transcript detection.

## Results

### Generation of a filtered set of fusion transcript predictions for CCLE cell lines

**Fig. 1:**
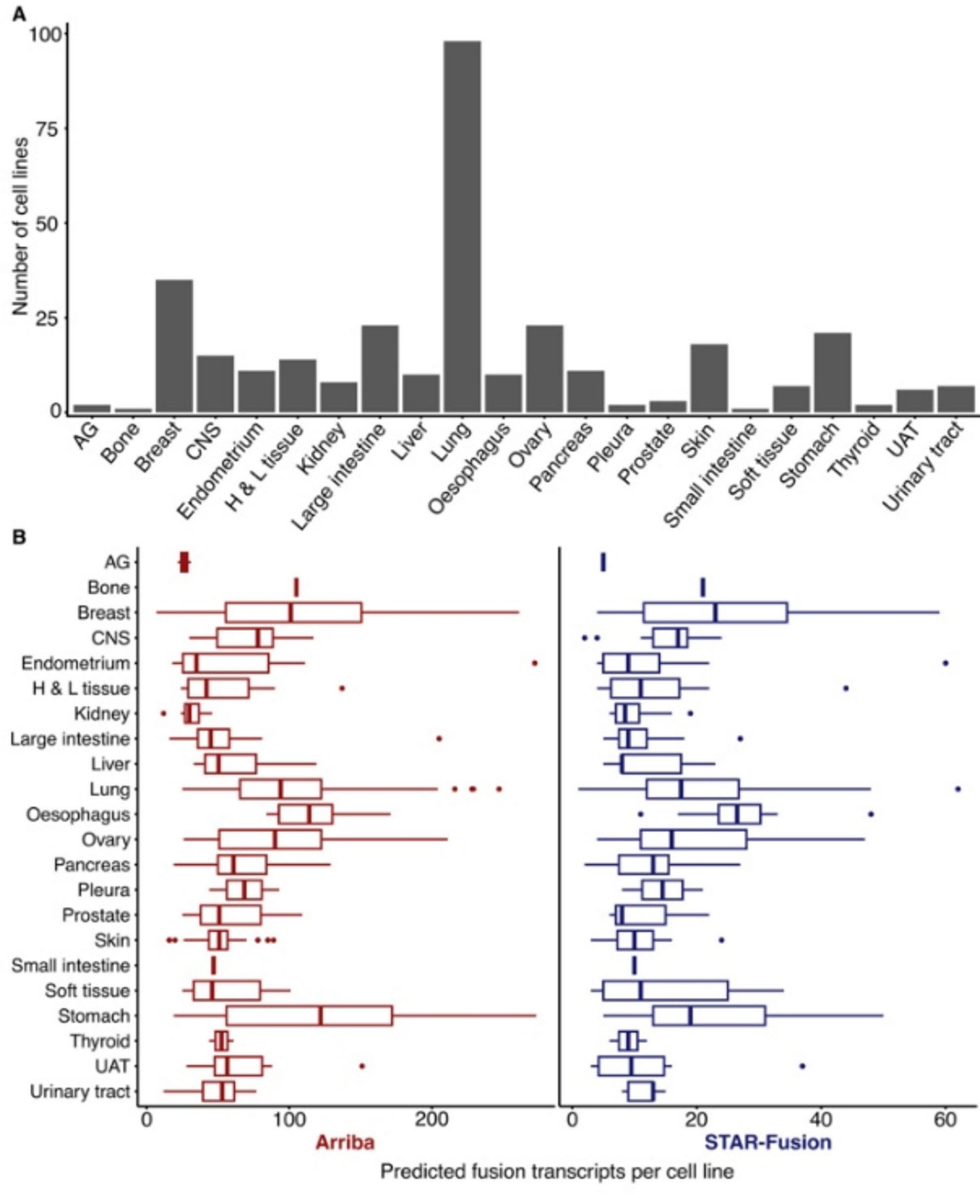
Tissue and cell line overview. **A** Cell lines per tissue type as represented in the dataset. **B** Predicted fusion transcripts per cell line and tissue type based on transcripts input to the validation pipeline. Tissue type abbreviations: autonomic ganglia (AG), central nervous system (CNS), haematopoietic and lymphoid tissue (H & L tissue), upper aerodigestive tract (UAT)

Within the publicly available CCLE datasets, we identified 328 cell lines from 22 different tissue types that had matched RNA-Seq and WGS data that could be used to create a resource of validated gene fusions. The number of individual cell lines varies considerably per tissue type with a median of 10 cell lines and a range of 1 to 98 cell lines (Fig. 1A). We used the RNA-Seq data as input for the two fusion transcript callers Arriba and STAR-Fusion. The initial numbers of reported events were 39,556 for Arriba and 6,123 for STAR-Fusion. We then performed a number of filtering steps to focus on fusion transcripts that could be used in the continued analysis. This included for example removal of other types of structural rearrangements and predictions with junctions in intergenic regions from the set of predictions (see Methods for details). Since the available WGS data were aligned to an older human genome assembly (hg19/GRCh37) we also used LiftOver [27] to convert fusion junction coordinates. Ultimately, 27,258 (69%) of the Arriba and 5,774 (94%) of the STAR-Fusion original predictions were used as input for the validation pipeline. The distribution across tissue types of these filtered fusion transcripts is shown in Fig. 1B. The overlap was 2,592 fusion transcripts with identical predicted fusion junctions, corresponding to 8% of the total set of unique predictions and 10 and 45% of the predictions from Arriba and STAR-Fusion, respectively.

### Creating a validated set of fusion transcripts with candidate genomic breakpoints

**Fig. 2:**
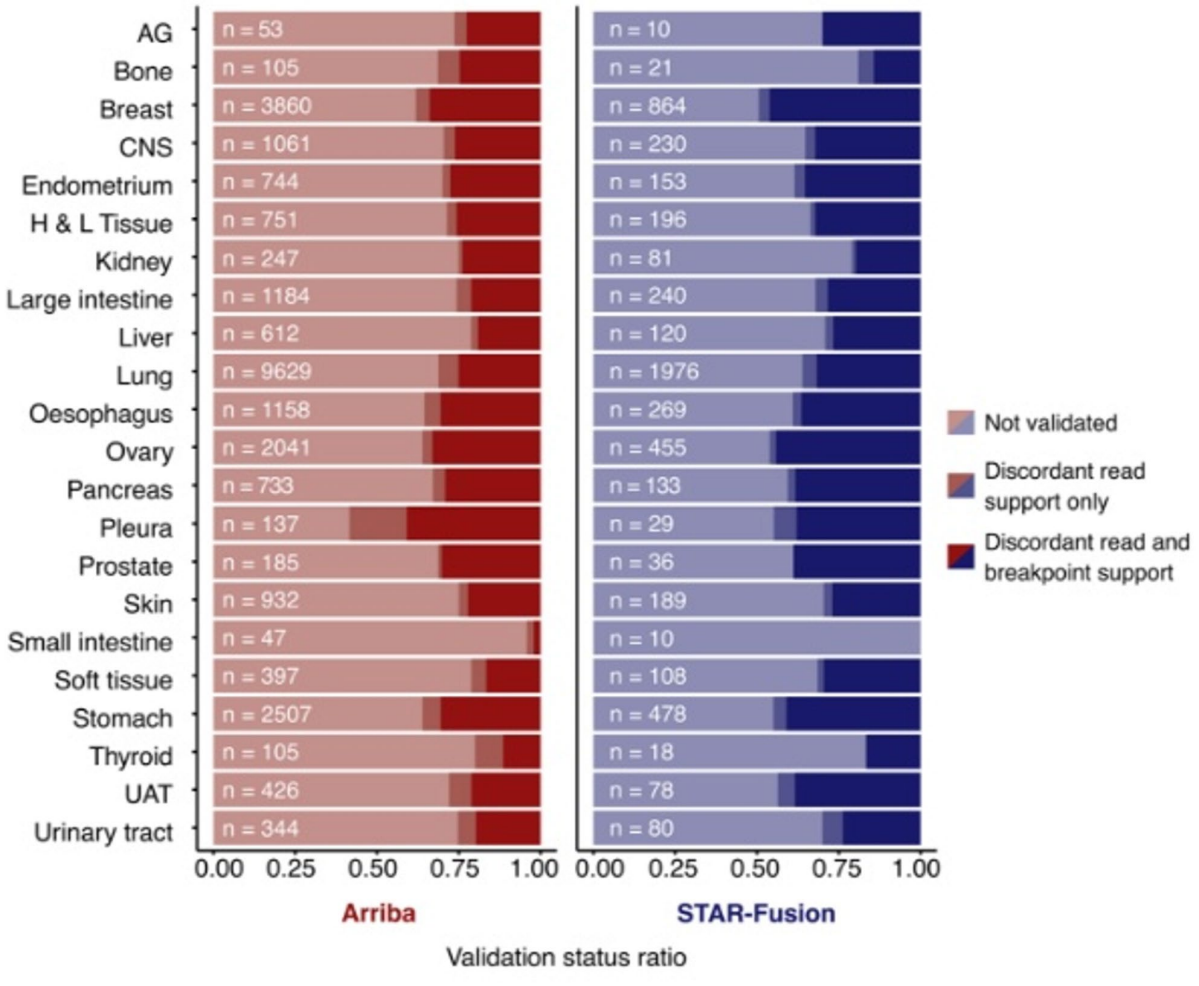
Fraction of fusion transcripts for each tissue type that were validated with support from discordant reads pairs only or discordant read pairs plus a candidate genomic breakpoint. Calculations were based on the filtered predictions which were input to the pipeline, represented by “n = ” on each bar. Tissue type abbreviations: autonomic ganglia (AG), central nervous system (CNS), haematopoietic and lymphoid tissue (H & L tissue), upper aerodigestive tract (UAT)

After generating this large set of predicted fusion transcripts, we employed our recently developed pipeline that uses WGS data for fusion gene validation [17]. The pipeline uses gene and fusion junction coordinates to parse matched WGS data for support in the form of discordant read pairs and candidate genomic breakpoints. Among the 27,258 predicted fusion transcripts from Arriba that were analysed with the pipeline, 8,753 (32%) could be validated at the DNA level with support from discordant read pairs; 7,428 or 85% of these also had an identified candidate genomic breakpoint (Fig. 2). The corresponding numbers for the 5,774 predicted fusion transcripts from STAR-Fusion were 2,244 (39%) validated with discordant read pairs and 2,045 or 91% of these with a candidate genomic breakpoint (Fig. 2). Of the 328 cell lines that were included in this study, 322 (98%) and 299 (91%) had validated fusions based on Arriba and STAR-Fusion predictions, respectively. The validation rate was clearly higher for fusion transcripts that were predicted by both software; 1,596 out of 2,592 fusion transcripts (62%), compared to 29 and 20% for fusions that were only predicted by Arriba or STAR-Fusion, respectively. This enrichment was significant with *p* < 2.2 × 10^− 16^ for both software (Fisher’s exact test).

Both Arriba and STAR-Fusion annotate their output with several additional features such as a prediction of the protein-coding status of the fusion transcript. The distributions of some of these features are included in Table 1. Arriba uses a ranking system for its confidence in a prediction with the scale low, medium, high (Table 1). Although a majority of the validated fusion transcripts (58%) were labelled as high confidence predictions, validated fusions were found across all confidence levels. In the set of fusion transcripts which were input to the validation pipeline, 9,519 predictions were assigned “high” confidence and 5,120 (54%) were validated, however we saw that 1,447 of the 10,140 (14%) “low” confidence predictions were also validated. Hard filtering of fusion transcript predictions from Arriba using only the indicated confidence level would therefore be insufficient to remove false positive predictions and lead to a loss of genuine fusion events.

**Table 1:** Summary of selected fusion transcript characteristics from Arriba and STAR-Fusion. Pre-pipeline fractions were calculated based on the number of transcripts input to the validation pipeline. Validated fractions were calculated based on fusions validated with discordant read support at minimum. An em dash (—) indicates that the programme did not output a comparable characteristic. Reading frame: A designation of “stop-codon” indicates a stop codon prior to the fusion junction [28] Transcription type: “Sense” are fusions where both partners were transcribed in the sense direction of the annotated genes. “Antisense” are fusions where one or both partners were transcribed in the antisense direction of the annotated genes. Prediction confidence: A ranking system provided by Arriba on the credibility of each prediction

	Arriba predictions		STAR-Fusion predictions	
	Pre-pipeline	Validated	Pre-pipeline	Validated
	*N* = 27,258	*N* = 8,753	*N* = 5,774	*N* = 2,244
*Reading frame*				
In-Frame	5,404 (0.20)	1,401 (0.16)	1,041 (0.18)	620 (0.28)
Out-of-Frame	10,197 (0.37)	4,291 (0.49)	940 (0.16)	612 (0.27)
Stop-codon	488 (0.02)	133 (0.02)	—	—
Unknown	11,169 (0.41)	2,928 (0.33)	3,793 (0.66)	1,012 (0.45)
*Transcription type*				
Sense	22,079 (0.81)	7,169 (0.82)	5,774 (1.00)	2,244 (1.00)
Antisense	5,179 (0.19)	1,584 (0.18)	0 (0.00)	0 (0.00)
*Prediction confidence*				
High	9,519 (0.35)	5,120 (0.58)	—	—
Medium	7,599 (0.28)	2,186 (0.25)	—	—
Low	10,140 (0.37)	1,447 (0.17)	—	—

### Fusions with observed alternatively spliced transcripts are more common among validated fusion genes

We then annotated fusion transcripts based on a number of different features, both to further characterize the fusions and to make the dataset more useful for the research community (Table 2). Since fusion gene pairs can appear as alternatively spliced transcripts within a given sample [29], we first identified candidates of possible alternative splicing. We considered a fusion as an alternative splicing candidate if the fusion gene pair was reported more than once by the same programme, in the same cell line, but with different fusion junctions. An alternative explanation in these cases could be the existence of multiple genomic breakpoints, for example, as a result of complex amplification events. Here, we have not attempted to determine the exact mechanism for individual events but simply label these cases as candidate alternative splicing.

**Table 2:** Annotation summary. Fractions were calculated based on fusions validated with discordant read support at minimum and after collapsing alternatively spliced fusion gene pairs into unique fusions in a cell line. If a fusion pair appeared multiple times (due to alternative splicing), but in at least one of those instances the specified situation applies, it was counted. Non-italicized rows represent fractions of the associated italicized row

	Arriba	STAR-Fusion
	*N* = 5,339	*N* = 1,834
*Possible promoter swapping*	0.12	0.13
*Involving kinases*	0.08	0.10
5’ only	0.60	0.52
3’ only	0.38	0.45
Both partners	0.02	0.03
*miRNA host genes*	0.08	0.08
5’ partner host only	0.58	0.49
3’ partner host only	0.41	0.50
Both partners host	0.01	0.01
*Matches to Mitelman database*	0.10	0.21
Exact tissue match	0.32	0.37
Related tissue match	0.01	0.01
Without tissue match	0.63	0.58
Unknown Mitelman tissue	0.04	0.05
*Matches to TumorFusions*	0.05	0.10
Exact tissue match	0.20	0.22
Related tissue match	0.02	0.03
Without tissue match	0.78	0.75
*Involving COSMIC CGC genes*	0.14	0.15
5’ only	0.64	0.59
3’ only	0.32	0.35
Both partners	0.04	0.06

So as not to skew results due to differences in the number of fusion transcripts per fusion gene pair, we report summary statistics for annotations such that each fusion gene pair is only counted once per cell line and prediction software. This brings the totals to 5,339 validated fusions from Arriba and 1,834 from STAR-Fusion. In the total set of fusion transcripts that were used as input to the validation pipeline, 25% of Arriba and 14% of STAR-Fusion collapsed gene pairs were candidates for alternative splicing. These percentages were higher within the validated set of fusion transcripts with 37% of Arriba and 19% of STAR-Fusion collapsed gene pairs representing an alternative splicing candidate from the input set. This association between the observation of candidate alternatively spliced fusion transcripts and pipeline validation was statistically significant for both Arriba and STAR-Fusion (*p* < 2.2 × 10^− 16^ and *p* = 3.5 × 10^− 13^, respectively, Fisher’s exact test).

### Cell lines frequently contain in-frame kinase fusions

**Fig. 3:**
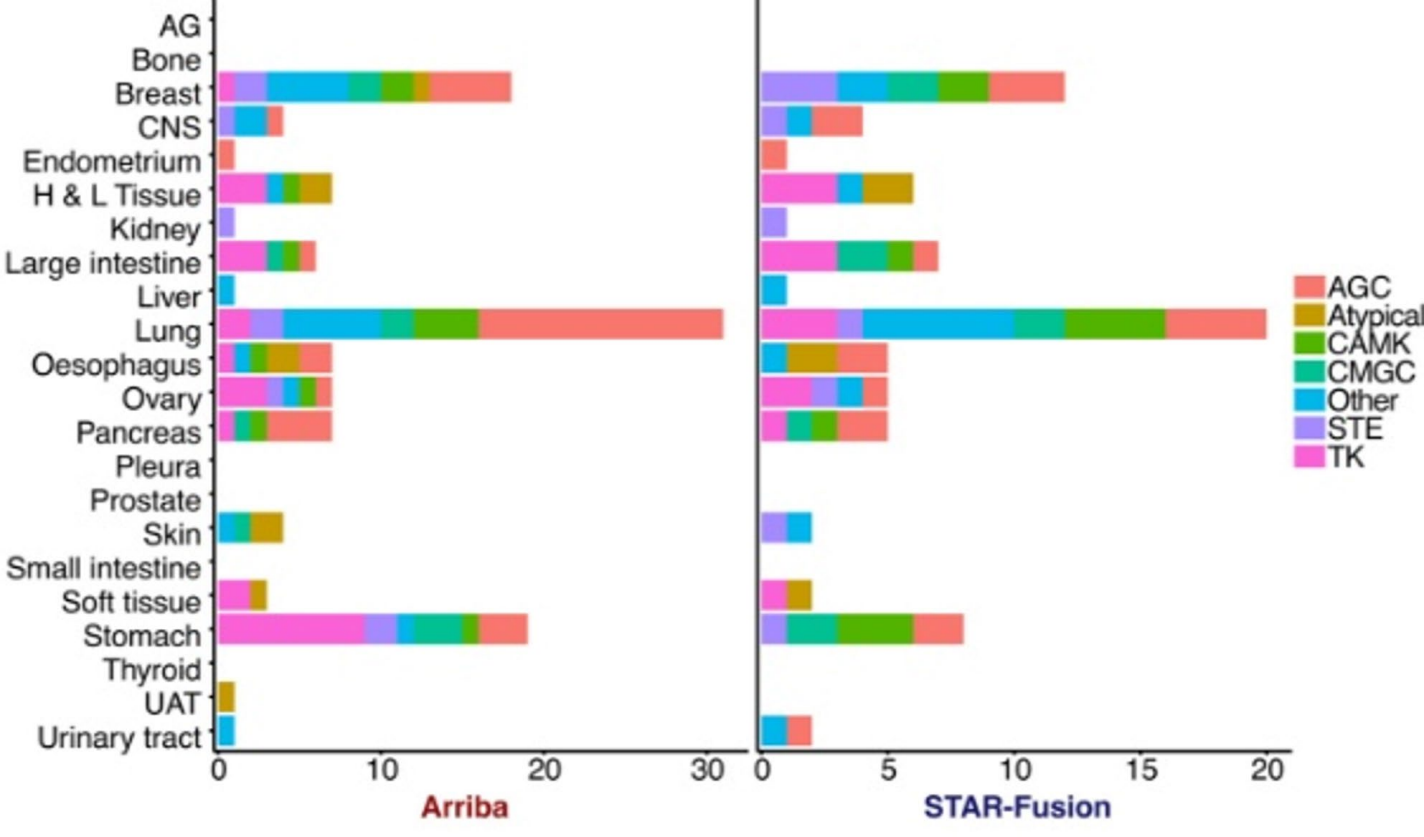
Kinase groups. Calculations based on validated fusions with in-frame kinases present as a 5’ or 3’ partner. Eligible fusion pairs are represented just once per cell line per programme. Kinase group abbreviations: kinase which belongs to the protein kinase A, G, or C families (AGC); Ca^2+^/calmodulin-dependent protein kinase (CAMK); cyclin-dependent kinase, mitogen-activated protein kinase, glycogen synthase kinase, and cyclin-dependent-like kinase (CMGC); kinase which belongs to the Ste7, Ste11, or Ste20 families (STE); and tyrosine kinase (TK). Tissue type abbreviations: autonomic ganglia (AG), central nervous system (CNS), haematopoietic and lymphoid tissue (H & L tissue), upper aerodigestive tract (UAT)

Fusions involving kinases can be compelling drug target candidates [2, 9], and we annotated for these cases with a special interest in in-frame kinase fusions. As shown in Tables 2, 8% of Arriba and 10% of STAR-Fusion validated fusions involve kinases. Among these, 114 Arriba and 78 STAR-Fusion validated kinase fusions were in-frame, corresponding to 27 and 42% of the validated kinase fusions, respectively. An additional 22 Arriba and 11 STAR-Fusion validated fusions represent possible promoter swapping events where the coding sequence of the 5’ gene is not included but its promoter controls the expression of the 3’ kinase partner. The remainder encompass both out-of-frame fusions and those whose reading frame could not be determined. Interestingly, 45 (39%) of the Arriba and 35 (45%) of the STAR-Fusion in-frame kinase fusions match a gene pair in the Mitelman database [22] or the TumorFusions dataset [7]. Fusions like these, that are found in both patient-derived cell lines and patient tumour samples, could be interesting candidates for functional studies and drug development. Validated in-frame kinase fusions were found in 80 (25%) of 322 cell lines with validated fusions based on Arriba predictions and in 63 (21%) of 299 cell lines with validated fusions based on STAR-Fusion predictions. The greatest number of kinase fusions were seen in lung, stomach, and breast tissue, which is consistent with the large number of cell lines representing these tissue types (Fig. 3). For both Arriba and STAR-Fusion, the AGC (protein kinase A, G, and C families) and tyrosine kinase (TK) groups were most represented across tissue types.

Reassuringly, both Arriba and STAR-Fusion identified previously detected fusion genes. Examples include *BCR::ABL1* in LAMA84, a blast phase chronic myeloid leukaemia (CML-BP) cell line [30] and *EML4::ALK* in NCI-H2228 (also known as H2228), a lung adenocarcinoma cell line [31]. The fusion events were supported by both discordant read pairs and genomic breakpoints. Other examples of recurrent in-frame kinase fusion partners include.

**Fig. 4:**
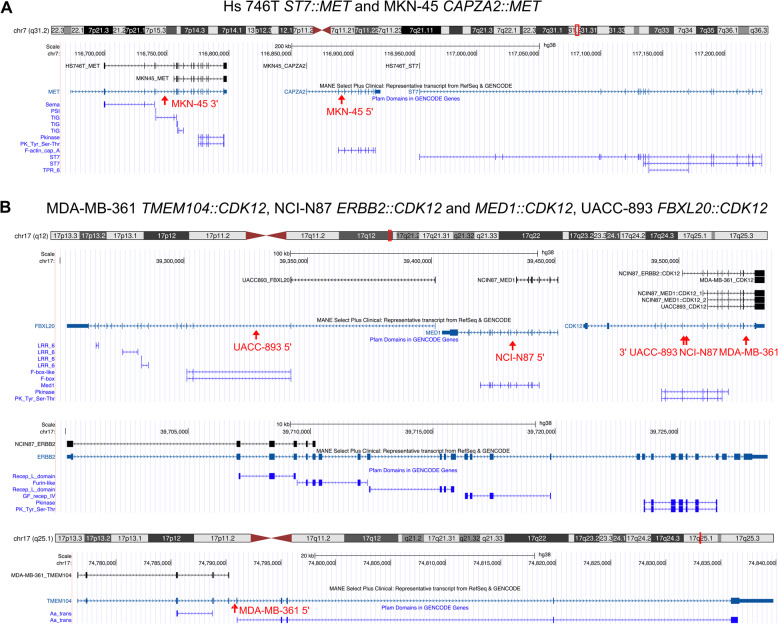
Examples of validated in-frame kinase fusions. A *MET* fusions with an intact kinase domain were found in the Hs 746T and MKN-45 cell lines. B *CDK12* fusions were found in the MDA-MB-361, NCI-N87 and UACC-893 cell lines. Black transcripts show exons included in the predicted fusion event and are labelled with cell line plus fusion partner. They end at the fusion junction for 5’ partners and start at the fusion junction for 3’ partners. Red arrows indicate genomic breakpoints, if detected, and red text labels the corresponding cell line. MANE reference transcript annotation (dark turquoise) and Pfam protein domains (pure blue) are included for reference. Image source: http://genome.ucsc.edu

*MET Proto-Oncogene*,* Receptor Tyrosine Kinase* (*MET*) in the gastric adenocarcinoma cell lines Hs 746T and MKN-45 (Fig. 4A). Both *ST7::MET* and *CAPZA2::MET* fusion transcripts include the full kinase domain. *MET* fusions are rare but have been found in many cancer types [32]. Similar to genomic amplification and splice site mutations leading to skipping of exon 14, *MET* fusions are a mechanism for upregulation of MET activity and could potentially be targeted with tyrosine kinase inhibitors. Interestingly, we found in-frame fusions involving *Cyclin Dependent Kinase 12* (*CDK12*) as a 3’ partner in three cell lines: MDA-MB-361, UACC-893 (both derived from breast carcinoma) and NCI-N87 (gastric carcinoma). CDK12 is a transcription-associated cyclin-dependent kinase with roles in regulation of transcription, splicing, and the DNA damage response [33]. It has been associated with both oncogenic and tumour-suppressive properties in different contexts [34]. The detected fusion transcripts lack the arginine/serine-rich (RS) motifs and nuclear localisation signals that are important for localisation of CDK12 in the nucleus. The kinase domain is partially retained in NCIN-87 and UACC-893 but absent in MDA-MB-361 (Fig. 4B). Information on promoter swapping and in-frame fusions involving kinases such as these can be used to plan functional studies and explore potential drug therapy targets.

### Gene fusions can lead to deregulation of small RNA expression

**Fig. 5:**
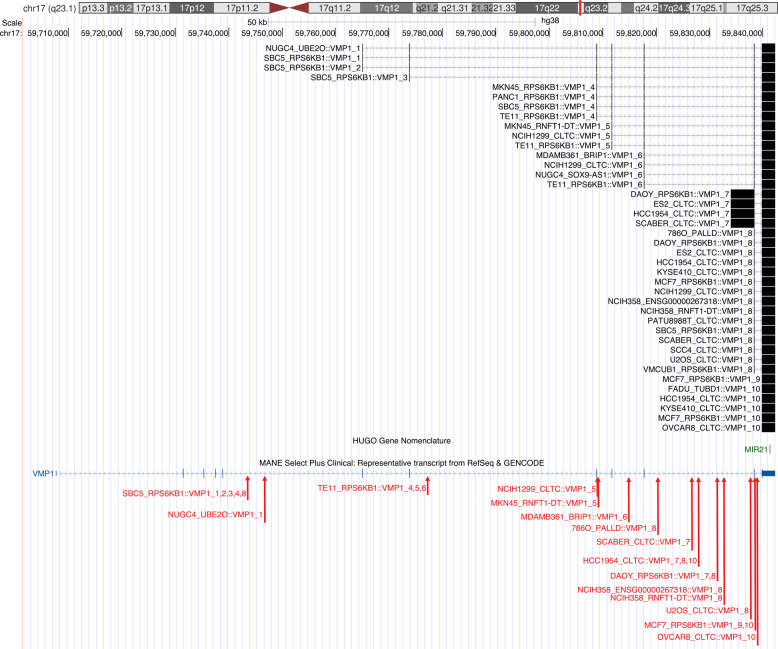
Visual representation of validated 3’ *VMP1* fusions which host *mir-21*. Black transcripts show the predicted 3’ partner fusion transcript and are labelled with the cell line plus 5’ fusion partner. They begin at the fusion junction for the 3’ *VMP1* partner and continue through the last *VMP1* exon. Red arrows indicate genomic breakpoints, if detected, and red text labels the fusion(s) which it corresponds with. HUGO annotation (green) displays the location of *mir-21* and the MANE reference transcript annotation (dark turquoise) is included for reference. Image source: http://genome.ucsc.edu

Small non-coding RNAs such as miRNAs are often encoded in the introns of longer transcripts and processed co-transcriptionally [35]. We annotated validated fusions for the presence of miRNA host genes and found that 8% of both Arriba and STAR-Fusion gene pairs involved at least one miRNA host gene (Table 2). Fusions involving miRNA host genes as 3’ partners will essentially be promoter swapping events, and can alter control of miRNA expression, regardless of the coding potential of the fusion transcript. We have proposed that this is a mechanism for deregulation of miRNA expression in cancer and coined the term “miRNA-convergent fusions” to describe recurrent gene fusions where multiple different 5’ partners are found together with a 3’ partner that is a miRNA host gene [36]. The most frequent miRNA host gene among 3’ partners was *VMP1*, host to the well-established oncogene *mir-21*, precursor of miR-21-5p [37]. Validated *VMP1* fusions were found in 22 cell lines from 11 different tissue types (Fig. 5). Among the 9 different 5’ partners, *CLTC* and *RPS6KB1* were the most common; both are located in the same frequently amplified region on chromosome 17q23.1. A particularly interesting example is a *BRIP1::VMP1* fusion in the MDA-MB-361 breast carcinoma cell line. The fusion of a tumour suppressor gene with a host gene of an oncogenic miRNA could lead to deregulation of target genes and further tumour proliferation. The most common miRNA hosted within a 5’ partner was *mir-1204* which is located in the *PVT1* gene; fusions were found in 20 cell lines from 6 different tissue types. Interestingly, *PVT1* has been implicated in cancer and overexpression of *mir-1204* is associated with tumour cell proliferation in breast cancer [38].

### Cell line fusions are detected in patient samples and involve genes causally implicated in cancer

For fusion-containing cell lines to be useful as models for functional studies and drug development, the fusion genes should ideally be candidate driver events that are recurrently found in cancer. We therefore annotated the set of validated fusion transcripts with information from the Mitelman database [22], the TumorFusions dataset [7] and the COSMIC CGC v102 list of genes “causally implicated in cancer” [23]. The Mitelman database has manually curated information about known gene fusions as well as the cancer and tissue types in which they were observed based on published literature. Comparison to the Mitelman database showed that 558 (10%) of Arriba and 380 (21%) of STAR-Fusion validated fusion gene pairs had been previously identified (Table 2).

The TumorFusions dataset contains fusions found in TCGA tumours and is based on analysis of RNA-Seq data where samples with matched DNA data underwent a validation process with structural variant calling for samples with WGS data and copy number data from SNP arrays [7]. Comparison to the TumorFusions dataset [7] showed that 291 (5%) Arriba and 181 (10%) STAR-Fusion validated fusion gene pairs were previously detected in tumour samples from TCGA (Table 2).

Comparison to the COSMIC CGC gene list showed that 745 (14%) Arriba and 281 (15%) STAR-Fusion validated fusions involve genes with a potential role as tumour drivers (Table 2). Interestingly, 147 (20%) of these Arriba and 91 (32%) of these STAR-Fusion validated fusions also matched a gene pair in the Mitelman database [22] or the TumorFusions dataset [7]. The remainder, 598 (80%) Arriba and 190 (68%) STAR-Fusion validated fusion gene pairs, did not match known gene fusions in the Mitelman and TumorFusions datasets. They could represent cases where the creation of a fusion gene is a rare mechanism for mutation of oncogenes and tumour suppressors.

Most gene fusions in cancer are believed to be passenger events [39]. In total, 120 unique fusion gene pairs involved a gene from the COSMIC CGC list and had been previously reported in the Mitelman and/or TumourFusions databases. Fusions which appear in our validated cell line dataset as well as in established fusion databases and are known to be causally implicated in cancer are of special interest when trying to identify novel driver events.

### Fusions4U: a web interface for fusion gene analysis

**Fig. 6:**
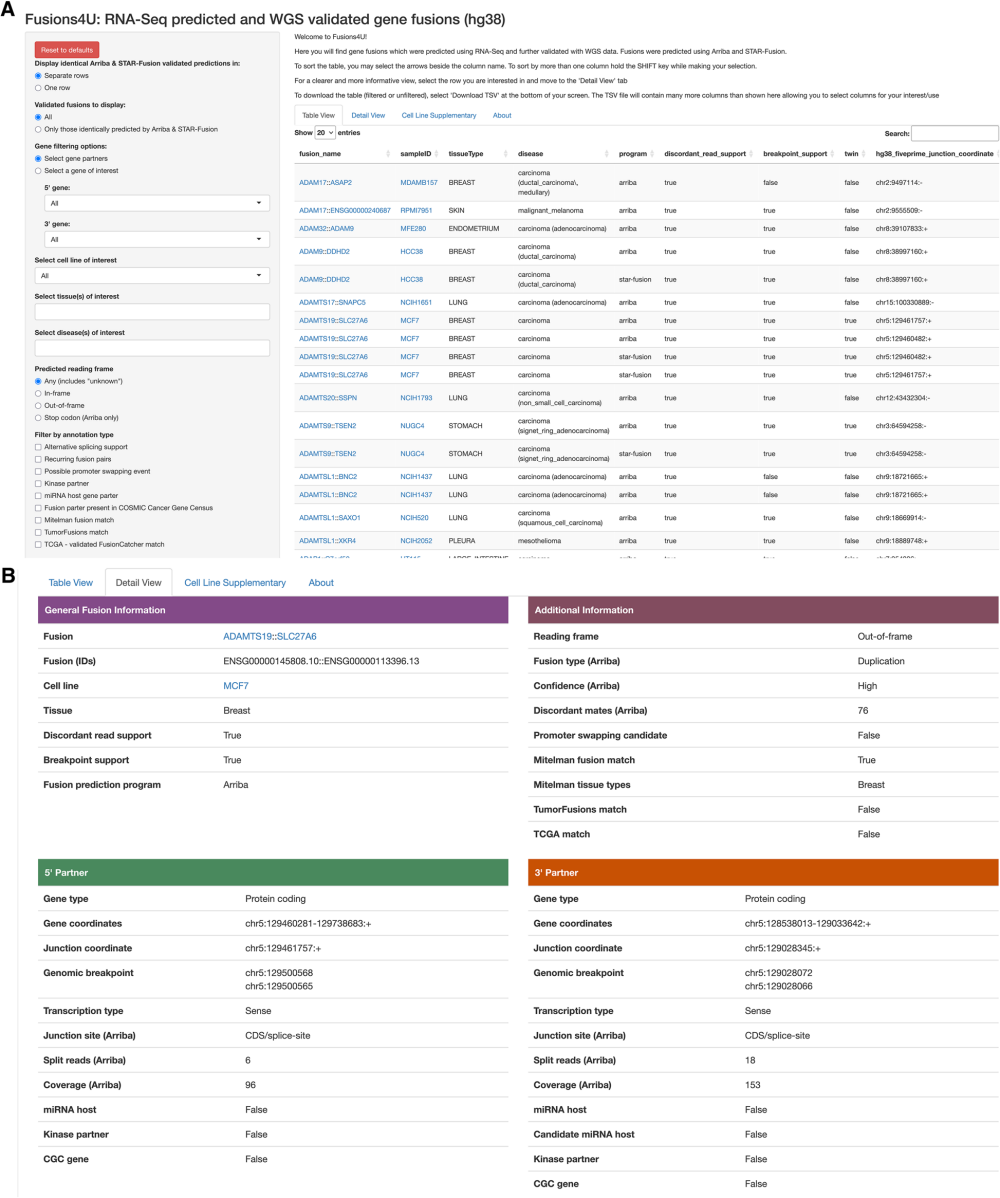
The Fusions4U web application. **A** In this view, the user can choose how to display information, filter, download a TSV of their filtered results, and select a fusion to view in more detail. **B** Based on the fusion selected in the table, this view provides a more concise way to interpret information in each column of the dataset

Our complete set of validated fusion transcripts is included here as supplementary material (Additional File 1) but to provide easy access to our results together with links to additional resources, we have also created the Fusions4U web interface (Fig. 6). It features a side panel with several options for filtering of fusion transcripts, as well as four tab panels for data display (Fig. 6A). In the “Table view” tab, the user can download a TSV-formatted (tab-separated values) text file with their filtered list of validated fusions or select a specific fusion which they would like to view more in-depth by clicking the “Detail view” tab (Fig. 6B). The “Cell Line Supplementary” tab includes information about the CCLE cell lines together with statistics for the RNA-Seq and WGS data, as well as validation statistics per programme. It also includes links to the Dependency Map (DepMap) portal [28] page for each cell line. The “About” tab has a table which details the contents of each column from the “Table view” as well as links to software and annotation sources.

### Case study: integrating validated fusions with PRISM drug screening data

**Fig. 7:**
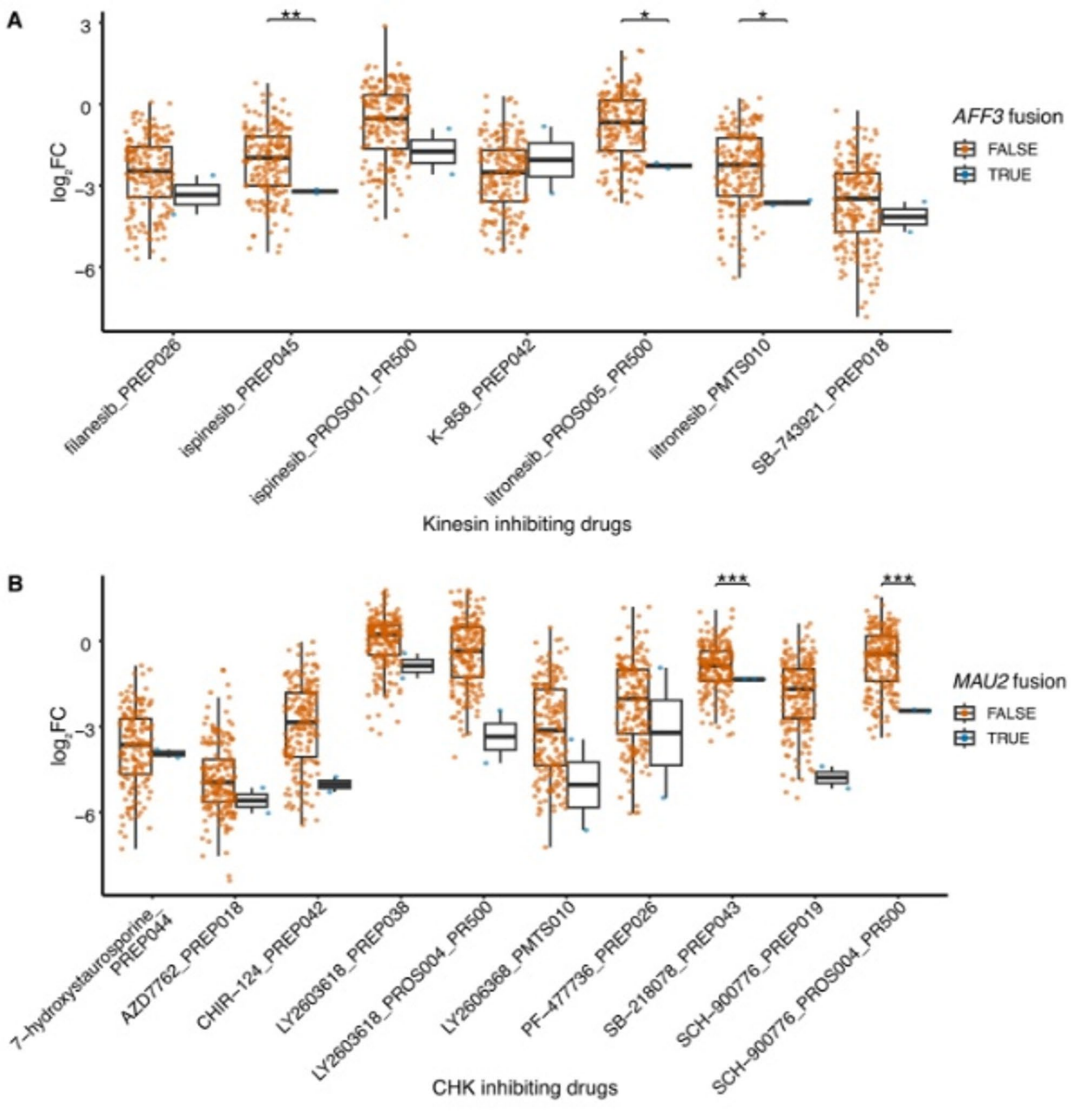
PRISM drug repurposing dataset analysis. Relative proliferation (log_2_ fold change) of cell lines with and without fusions in response to drugs with a common mechanism of action. **A** Cell lines with *AFF3* fusions (GAMG and HCC44) are sensitive to kinesin-inhibiting drugs. **B** Cell lines with *MAU2* fusions (MKN7 and SKUT1) are sensitive to CHK-inhibiting drugs. The x-axis labels include both drug and plate name. Data were plotted for a drug concentration of 2.5 µM, except for litronesib_PROS005_PR500 and PF-477736_PREP026, which were 0.16 and 0.60 µM, respectively. Statistical significance was determined by Welch’s *t*-test for each possible combination of fusion partner genes and drugs across all cell lines; the resulting p-values were adjusted for multiple testing using the Benjamini-Hochberg procedure (* p_adj < 0.05, ** p_adj < 0.01, *** p_adj < 0.001)

The DepMap portal [28] hosts a wealth of information for cell lines in the CCLE. This includes several types of omics data as well as results from large-scale screening experiments using libraries for RNA interference (RNAi), CRISPR knockout and drug response analysis. To demonstrate how our gene fusion database can be combined with existing data for the CCLE cell lines, we integrated information about validated fusions with drug screening data from the PRISM drug repurposing dataset [24, 26]. For each gene present in an in-frame or candidate promoter swapping fusion, cell lines were grouped by presence or absence of a fusion involving that gene and then tested for differences in log_2_ fold change for cell proliferation in response to treatment with a drug compared to vehicle (DMSO) control. All gene fusion partners with an in-frame or candidate promoter swapping fusion in at least two cell lines present in the PRISM drug repurposing dataset (202 unique genes) were tested for a significant difference in proliferation for all drugs (1052 unique compounds) using a two-sided Welch’s *t-*test followed by correction for multiple testing using the Benjamini-Hochberg procedure. The results are included in Additional File 2. Two examples where gene fusions are associated with response to specific drugs are shown in Fig. 7 and as supplementary information (Additional File 3, Figures S1-S3). While analyses like this do not prove that a fusion causes sensitivity to a specific drug, they can be used to generate testable hypotheses for functional studies and validation experiments.

## Discussion

Here we have created Fusions4U, an online resource of annotated fusion transcripts with WGS data validation for 328 cell lines from the CCLE. Available annotation includes information from the two fusion callers Arriba and STAR-Fusion such as fusion junction coordinates, read support and reading frame, as well as additional data on alternative splicing, candidate genomic breakpoints, kinases, miRNA host genes, known fusions from the Mitelman and TumorFusions databases, and genes causally linked to cancer from the COSMIC CGC. A wide range of genomic and functional data are available for CCLE cell lines through the DepMap portal [24, 28]. As we exemplify using drug screening data, the information about gene fusions that is provided here can be combined with external data to help design experiments for functional studies and drug development. Our fusion transcript validation pipeline identifies candidate genomic breakpoints that can be used to design primers for experimental validation or in analyses of the mechanisms that generate fusion genes. The Fusions4U resource also represents a large collection of fusion transcripts with DNA-level support from WGS data that can be used in evaluation of fusion transcript prediction software.

Gene fusions are a common type of genomic aberration in cancer [18]. Some are tumour driver mutations that are clinically important as drug targets [18]. They can also be used as diagnostic criteria [11] and as biomarkers for therapy response or cancer relapse in liquid biopsies [40]. The large clinical and scientific interest in gene fusions has led to the development of many different fusion transcript prediction software that can be used to analyse RNA-Seq data from cancer samples. In this study, we used Arriba [41] and STAR-Fusion [42], two established fusion callers that have performed well in published evaluations [14, 42]. At the time of writing, Arriba fusion calls are available in the latest (25Q2) release of the DepMap portal [24, 28, 43], but without additional validation or external annotation.

In our dataset, Arriba and STAR-Fusion provide complementary information with Arriba predicting a larger number of validated fusion transcripts than STAR-Fusion (8,753 vs. 2,244) but with a lower validation rate (32% vs. 39%). Although fusion transcripts jointly predicted by both software have a significantly higher validation rate (62%), these 1,596 validated fusion transcripts only represent a minority of the total validated set. The relatively low validation rate and the limited overlap between fusion callers emphasise the value of combining fusion transcript prediction with analysis of matched WGS data. This also demonstrates a problem with using recurrent prediction by multiple tools (so-called ensemble predictions) as a filtering criterion; it leads to retention of false positive predictions and loss of true gene fusion events. Arriba includes a feature which allows users to provide structural variant calls from WGS data to search for genomic breakpoints. We have not evaluated this feature since we wanted to use a consistent validation method for results from both Arriba and STAR-Fusion. Our published tool for validation of fusion transcripts in WGS data [17] only uses aligned sequence data for relevant genomic regions, decreasing the data storage space needed and facilitating analysis of this large sample set. The TumorFusions database also used structural variant calls as a form of validation [7] but there are clear advantages with using a method that utilizes stricter criteria, identifying discordant read pairs and candidate genomic breakpoints at the DNA level.

Many kinase fusions act as tumour drivers and can be targeted with kinase inhibitors which makes them attractive drug targets [44]. Examples include the *BCR::ABL1* gene fusion in patients with CML [2] and *ALK* fusions (e.g. *EML4::ALK*) in non-small-cell lung cancer [9]. *NTRK1* fusions are found in cancers across many different tissues, and the tyrosine kinase inhibitor larotrectinib is now used in patients positive for fusions involving *NTRK1* [45]. *FGFR2* fusions are being studied as targets for therapy and medications such as futibatinib and pemigatinib have recently been approved as an additional form of therapy for patients with *FGFR2* fusions [46]. Here we have shown examples of recurrent in-frame fusion genes with the kinases *CDK12* and *MET* as 3’ partners, fusions that have previously also been found in tumour samples [32, 33]. Another interesting example is *VMP1*, host gene to the miRNA *mir-21*, which is found as a 3’ fusion partner in nearly 7% of the analysed cell lines. This miRNA is a known tumour driver, and its upregulation can lead to chemo- and radiotherapy resistance [47].

The large set of fusions that we present here can be further analysed with focus on kinases or other interesting genes. The accompanying annotation can be used to select fusions present in the Mitelman and TumorFusions databases that have been detected in patient samples, and fusions involving genes linked to cancer from the CGC list. The combination of our data with information that is publicly available for CCLE cell lines, such as drug and gene knockdown response, can help identify clinically relevant fusions and candidate driver events for further studies.

## Conclusions

We have combined matched RNA-Seq and WGS data to create a resource of validated and annotated gene fusions from 328 cell lines from the CCLE. The dataset is included here as supplementary information (Additional File 1) and is available from the Fusions4U web application (https://fusions4u.serve.scilifelab.se/app/fusions4u). This dataset is a useful resource for design of experiments in functional studies and drug development, especially when combined with the wealth of information that is publicly available for the CCLE cell lines. The large collection of validated fusion transcripts with candidate genomic breakpoints can also be used in the development and evaluation of bioinformatic tools for fusion transcript detection.

### Methods

**Fig. 8:**
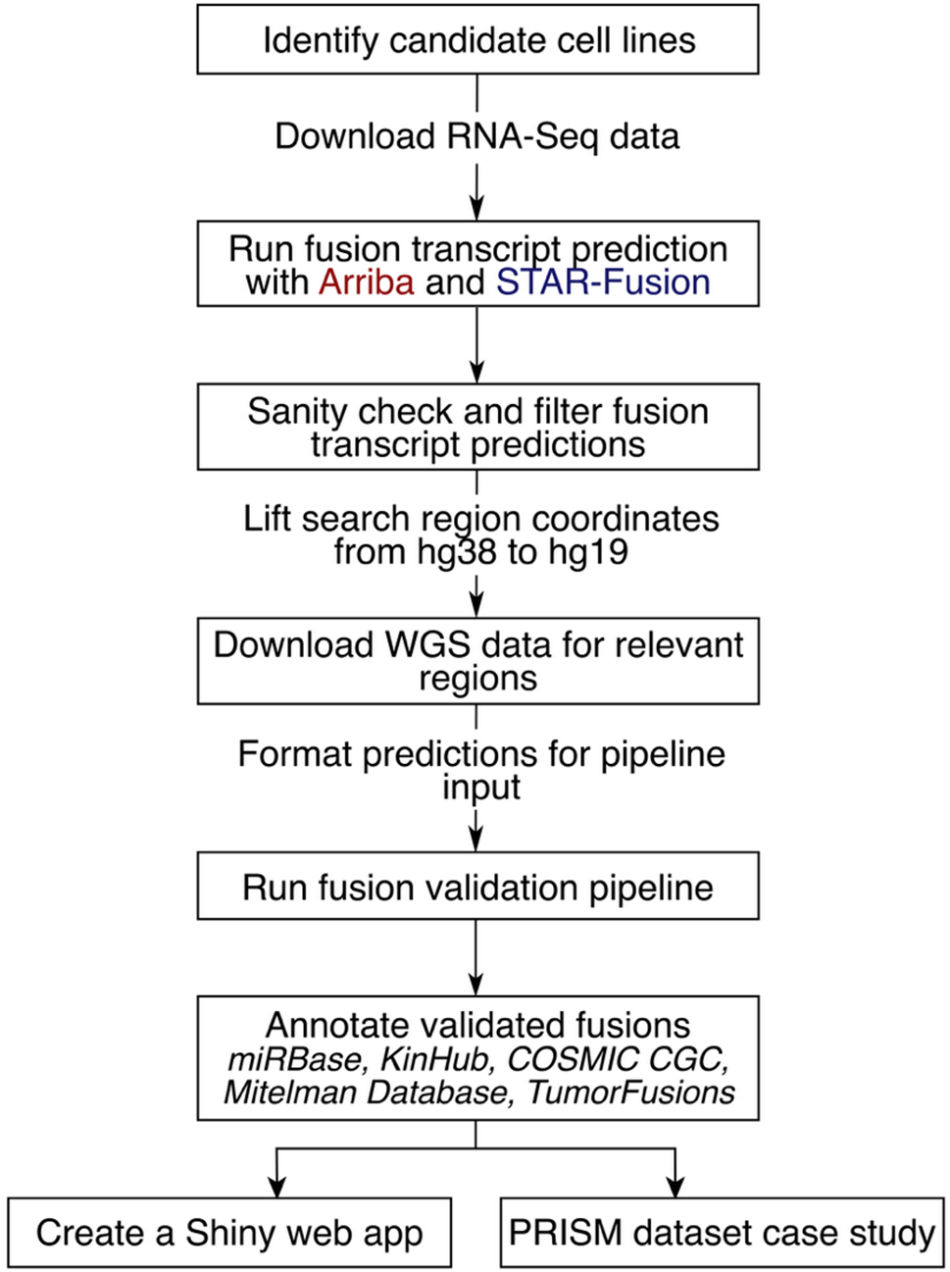
Schematic overview of the analyses

A graphical overview of the analyses is included in Fig. 8.

### Identification of candidate cell lines and RNA-Seq data download

We identified 328 cancer cell lines from the CCLE (accession PRJNA523380) that had matched RNA-Seq and WGS data available from the Sequence Read Archive (SRA) of The National Center for Biotechnology Information (NCBI) [48]. Paired-end Illumina HiSeq RNA-Seq FASTQ files were downloaded via individual SRA run accession numbers with the SRA Toolkit (v24.7.1).

### Fusion transcript prediction with arriba and STAR-Fusion

The human reference genome GRCh38.p14/hg38.14 from the UCSC Genome Browser [49] and GENCODE v44 transcript annotation file [50] were used for alignments and fusion transcript prediction. For Arriba, we first generated alignment files using STAR: RNA-Seq aligner v2.7.11b [51] and then ran Arriba v2.4.0 [41] with the recommended parameters for increased sensitivity and specificity, utilizing the provided known fusions and blacklist files and a protein domain annotation file generated via the UCSC Data Integrator [52]. We ran STAR-Fusion v1.13.0 [42] with the provided pre-built Trinity Cancer Transcriptome Analysis Toolkit (CTAT) library for GRCh38 with GENCODE v44 annotations [53] using the recommended parameters plus inclusion of reading frame prediction for fusion transcripts.

The output from Arriba and STAR-Fusion was filtered to remove fusion transcripts where the fusion junction was located in an intergenic region, outside the genomic coordinates of a partner gene. For Arriba we also excluded predictions corresponding to other types of structural rearrangements than gene fusions involving two distinct genes and predictions where the transcribed strand of at least one partner could not be predicted. For STAR-Fusion we excluded immunoglobulin (IG) superlocus fusion transcripts since the IG superlocus genes were only present in the pre-built CTAT genome resource library but not in the GENCODE v44 annotation file.

### WGS data download

WGS data aligned to hg19 and available for SRA accession number PRJNA523380 was used for fusion transcript validation. For each cell line we identified all genes involved in fusion transcripts and added 2 kilobase pair (kb) up- and downstream of the annotated start and end coordinates to generate individual lists of search regions. The genomic coordinates were then converted from hg38 to hg19 using the UCSC LiftOver tool [27] and used to retrieve read pairs mapping to the relevant regions with sam-dump. The resulting BAM (binary alignment map) files were merged for individual cell lines. Sequencing depth was calculated using mpileup from SAMtools v1.21.

### Validation of fusion transcripts

WGS data was analysed using our published pipeline, which searches fusion- and partner gene-specific regions for discordant read pairs and candidate genomic breakpoints to support fusion transcripts [17]. The input is a TSV file with the required columns of sample identifier, 5’ search region, 3’ search region, a path to the WGS BAM file, and a unique numerical identifier for each fusion in the file. The search area for the 5’ partner is defined as the region between the fusion junction and gene end and for the 3’ partner as the region between the gene start and the fusion junction. As part of the pipeline, a 500 bp (bp) flank up/downstream of the fusion junction and 2 kb flank up/downstream of the gene start/end is automatically added to the provided search coordinates [17]. Coordinates were converted from hg38 to hg19 using the UCSC LiftOver tool [27] and checked for consistency with hg19 gene coordinates. Fusion transcripts are considered “discordant read supported” if the pipeline finds discordantly mapped read pairs where the two reads map within the search regions of the two partner genes [17]. The pipeline then searches for genomic breakpoints with the use of soft-clipped reads. Since soft-clipping is a way to mark portions of a read which do not align to the reference, a soft-clipped sequence of high quality in the defined search region and near to a discordant read pair could indicate a genomic breakpoint. Candidate soft-clipped sequences are checked for valid orientation (informed by both the fusion partner and the strand), filtered, and checked for alignment near the corresponding discordant read’s mate. If the soft-clipped sequences “pass” each of these steps, then the fusion is also considered “breakpoint supported”. Identified candidate genomic breakpoints were converted from hg19 to hg38 using the UCSC LiftOver tool [27] and tested for consistency with gene and fusion coordinates. Discordant read support was here seen as sufficient evidence to consider a fusion transcript validated even when a genomic breakpoint could not be identified.

### Annotation of validated fusion transcripts

Gene fusions were annotated for instances of possible alternative splicing, i.e. gene pairs with more than one identified fusion transcript with different fusion junctions, predicted by the same software, and predicted within the same cell line. Fusions meeting all criteria were provided with identifiers (IDs) unique to that fusion and cell line combination. Possible promoter swapping events were defined as fusion transcripts where the 5’ fusion junction appeared before the start of the coding region of the 5’ partner gene according to annotation from GENCODE v44 [50].

When using external datasets to annotate for specific partners in a fusion (i.e. miRBase, KinHub, COSMIC CGC) or fusion pairs (i.e. Mitelman Database and TumorFusions), we did not annotate fusion partners or pairs which were predicted to exhibit alignment antisense to annotated genes. All fusions involving kinases were annotated using the KinHub list of human kinases [20] using the UniProt Retrieve/ID mapping webtool [54] to pull gene IDs corresponding with UniProt IDs. Annotation from miRBase v22 [21] was used to identify miRNA host genes among fusion partners, including genes where the hairpin precursor was located between the start and end coordinates and candidate hosts for miRNAs located within 2 kb downstream of the 3’ end. We only included cases where the miRNA was located within the fusion transcript.

To annotate for matches to existing fusion datasets, we used the Mitelman database [22] and the TumorFusions dataset [7]. For the Mitelman database, fusion pairs were matched by gene symbols and matches were further annotated for tissue type. Similarly, matching for TumorFusions was based on gene symbols with further annotation for tissue type according to the specified cancer (e.g. breast invasive carcinoma was assigned “breast” tissue), but with the addition of annotating on their reported fusion junction coordinates as well. We also used the COSMIC CGC (GRCh38, v102) [23] to annotate fusions which involved these genes of interest.

Lastly, we annotated for identical fusions predicted by both Arriba and STAR-Fusion. To be considered an identical fusion, two fusions had to have identical fusion junctions, be predicted by different software within the same cell line, and have identical annotations (e.g. reading frame, candidate genomic breakpoint positions when breakpoint supported by the validation pipeline, miRNA host, kinase, etc.). These identical fusions, or “twins”, were also provided with a unique twin ID for easy identification in the dataset.

### Creation of the web interface using R Shiny

For an accessible way to browse the dataset, we created an app with shiny (v1.10.0) [55] in R (v4.3.3) [56] which allows users to easily filter, sort, view, and download the fusion dataset. It also includes links to online resources for more information about fusion partner genes and cell lines.

### Analysis of the PRISM drug repurposing dataset

We downloaded data for the PRISM secondary drug screening which includes 1,448 drugs with positive response in the primary screening that were rescreened in triplicate using eight concentrations per drug ranging from 610 pM to 10 µM [24, 26, 28]. For the 198 cell lines which overlapped between our dataset and the PRISM secondary screening, we only kept measurements for drug concentrations tested in at least 80% of cell lines. One concentration was selected per drug and compound plate (treatment) by calculating the mean concentration, the differences between individual concentrations and the mean, and then choosing the concentration with the smallest absolute difference from the mean. This resulted in 1,496 treatments (1,340 unique drugs) which decreased to 1,153 treatments (1,052 unique drugs) after removing drugs without information about the mechanism of action and drug category (chemotherapy, targeted cancer, noncancer). We then identified 202 fusion partner genes that occurred in in-frame or candidate promoter swapping fusions in at least two different cell lines. All combinations of partner genes and treatments were tested for significant differences between cell lines with and without a fusion involving the specific gene in log_2_ fold change for proliferation compared to vehicle (DMSO) control using a two-sided Welch’s *t-*test followed by correction for multiple testing using the Benjamini-Hochberg procedure.

## Supplementary Information

Additional file 1. Complete set of validated and annotated gene fusions

Additional file 2. Significant (p_adj) t-test results from analysis of the PRISM drug repurposing dataset. 

Additional file 3. Additional figures from analysis of the PRISM drug repurposing dataset

## Data Availability

The sequencing data analysed in the current study are available from the NCBI SRA, accession number PRJNA523380. The code is available from the following GitHub repository: https://ithub.com/ariannaa7/Fusions4U. The Fusions4U web application: https://usions4u.serve.scilifelab.se/app/fusions4u.

## Abbreviations

AG: Autonomic ganglia
AGC: Group encompassing kinases of protein kinase A, G, and C families
BAM: Binary alignment map
bp: Base pair
CAMK: Group encompassing Ca2+/calmodulin-dependent protein kinases
CCLE: The Cancer Cell Line Encyclopedia
CMGC: Group encompassing cyclin-dependent kinases, mitogen-activated protein kinases, glycogen synthase kinases, and cyclin-dependent-like kinases
CML: Chronic myeloid leukaemia
CML-BP: Chronic myeloid leukaemia–blast phase
CNS: Central nervous system
COSMIC CGC: The Catalogue of Somatic Mutations in Cancer, Cancer Gene Census
CRISPR: Clustered regularly interspaced short palindromic repeats
CTAT: Trinity Cancer Transcriptome Analysis Toolkit
DepMap: Dependency Map
hg/GRCh: Genome Reference Consortium Human
H & L tissue: Haematopoietic and lymphoid tissue
ID: Identifier
IG: Immunoglobulin
kb: Kilobase pair
miRNA: microRNA
Mitelman database: Mitelman Database of Chromosome Aberrations and Gene Fusions in Cancer
NCBI: The National Center for Biotechnology Information
PRISM: Profiling relative inhibition simultaneously in mixtures
RNA-Seq: RNA sequencing
RNAi: RNA interference
RS: Arginine/serine
SRA: Sequence Read Archive
STE: Group encompassing kinases which belong to the Ste7, Ste11, and Ste20 families
TCGA: The Cancer Genome Atlas
TK: Tyrosine kinase
TKI: Tyrosine kinase inhibitor
TSV: Tab-separated values
UAT: Upper aerodigestive tract
UCSC: University of California, Santa Cruz
WGS: Whole-genome sequencing
