# Supplementary material for: Fusions4U: a resource of validated and annotated gene fusions in 328 cancer cell lines
Source: BMC Cancer. 2025 Dec 20;25:1908. doi: 10.1186/s12885-025-15441-w (PMC12752044; doi:10.1186/s12885-025-15441-w)

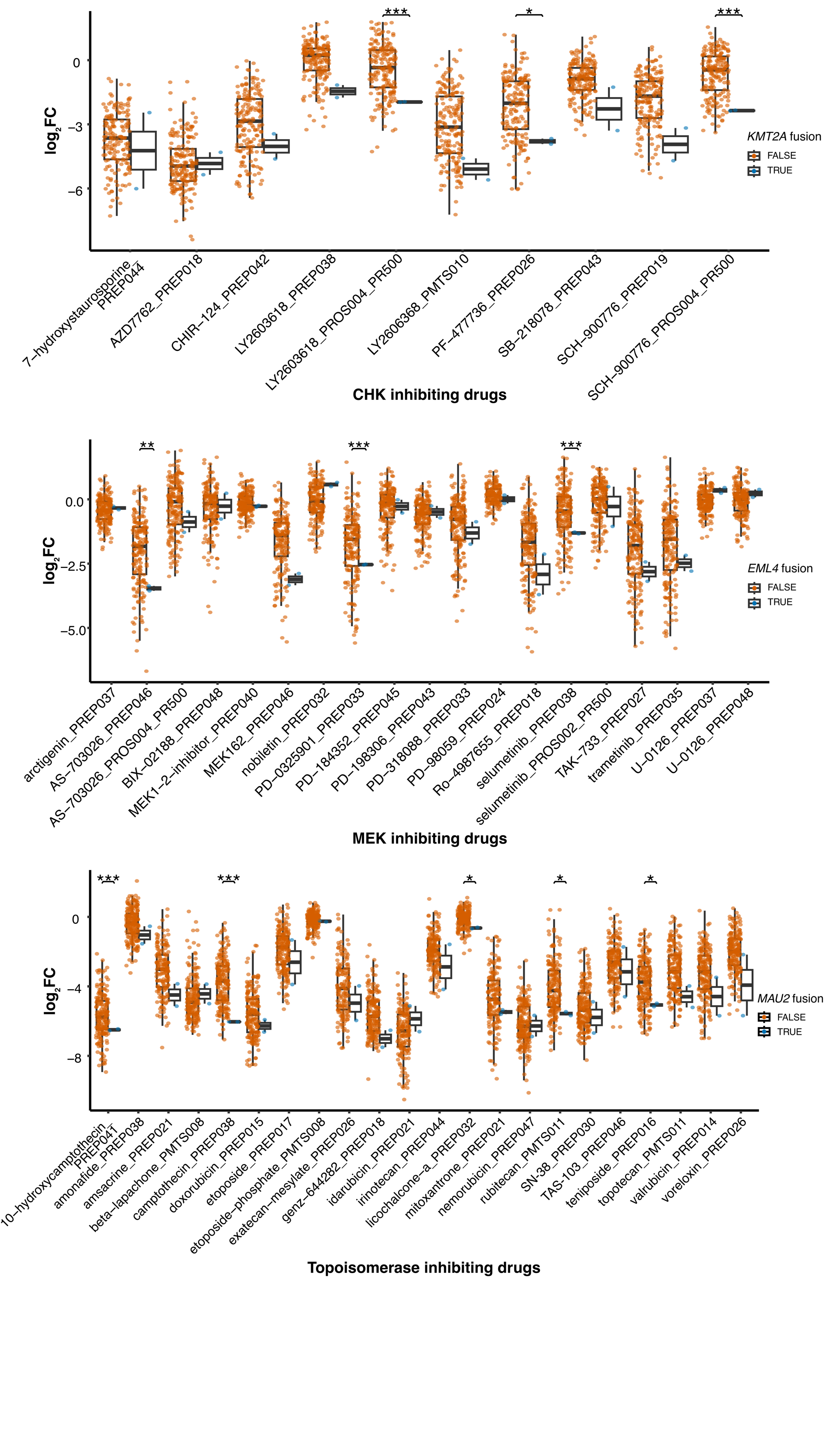


**Figure S1.** PRISM drug repurposing dataset analysis. Relative proliferation (log_2_ fold change) of cell lines with and without fusions in response to drugs with a common mechanism of action. Cell lines with KMT2A fusions (HCC38 and CAOV3) are sensitive to CHK-inhibiting drugs. The x-axis labels include both drug and plate name. Data were plotted for a drug concentration of 2.5 µM, except for PF-477736_PREP026 which was 0.60 µM. Statistical significance was determined by Welch’s t-test for each possible combination of fusion partner genes and drugs across all cell lines; the resulting p-values were adjusted for multiple testing using the Benjamini-Hochberg procedure (* p_adj < 0.05, ** p_adj < 0.01, *** p_adj < 0.001).


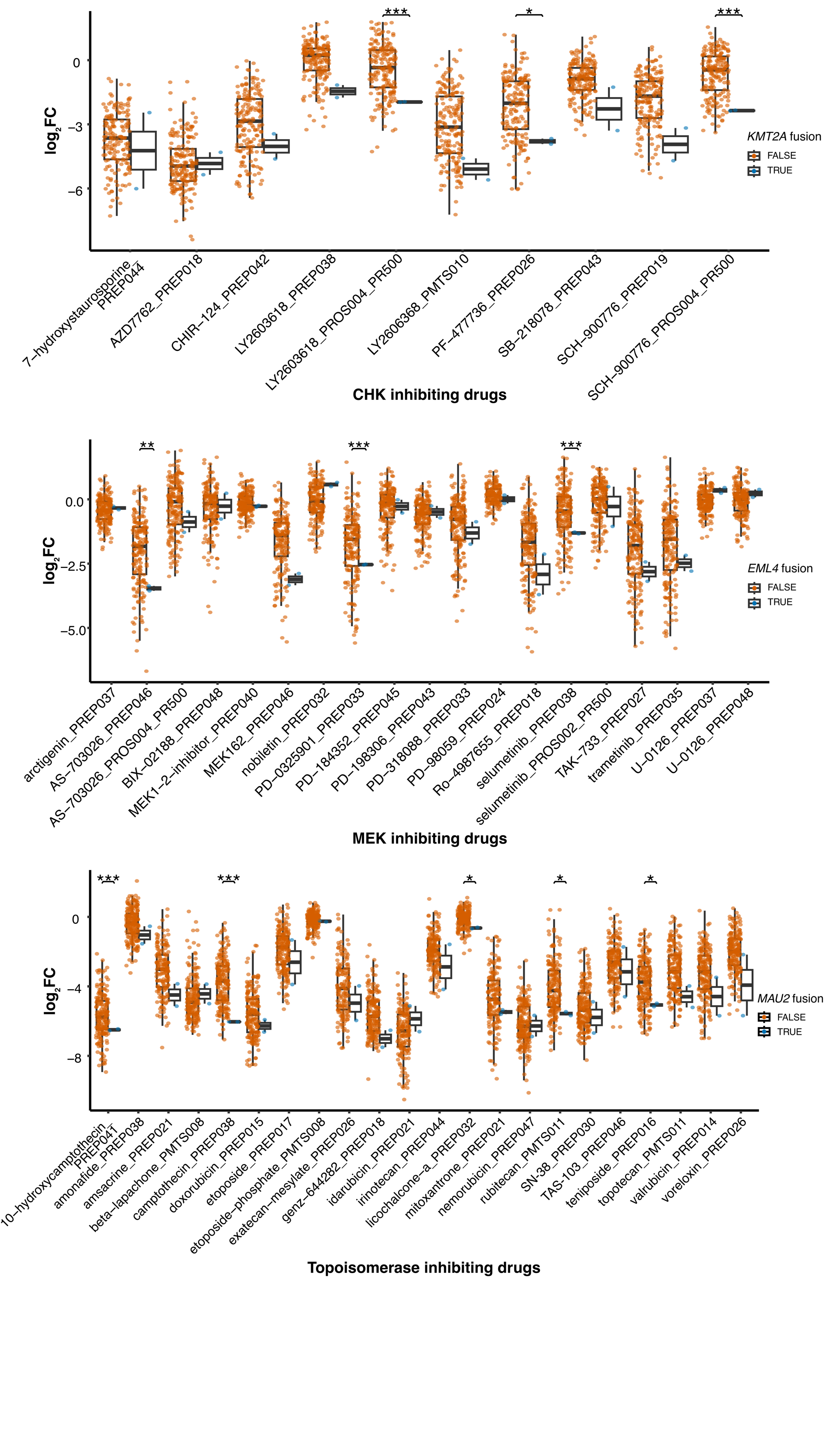


**Figure S2.** PRISM drug repurposing dataset analysis. Relative proliferation (log_2_ fold change) of cell lines with and without fusions in response to drugs with a common mechanism of action. Cell lines with EML4 fusions (HDQP1 and NCIH2228) are sensitive to MEK-inhibiting drugs. The x-axis labels include both drug and plate name. Data were plotted for a drug concentration of 2.5 µM. Statistical significance was determined by Welch’s t-test for each possible combination of fusion partner genes and drugs across all cell lines; the resulting p-values were adjusted for multiple testing using the Benjamini-Hochberg procedure (* p_adj < 0.05, ** p_adj < 0.01, *** p_adj < 0.001).

**Figure S3.** PRISM drug repurposing dataset analysis. Relative proliferation (log_2_ fold change) of cell lines with and without fusions in response to drugs with a common mechanism of action. Cell lines with MAU2 fusions (MKN7 and SKUT1) are sensitive to topoisomerase-inhibiting drugs. The x-axis labels include both drug and plate name. Data were plotted for a drug concentration of 2.5 µM, except for exatecan-mesylate_PREP026, which was 0.63 µM. Statistical significance was determined by Welch’s t-test for each possible combination of fusion partner genes and drugs across all cell lines; the resulting p-values were adjusted for multiple testing using the Benjamini-Hochberg procedure (* p_adj < 0.05, ** p_adj < 0.01, *** p_adj < 0.001).


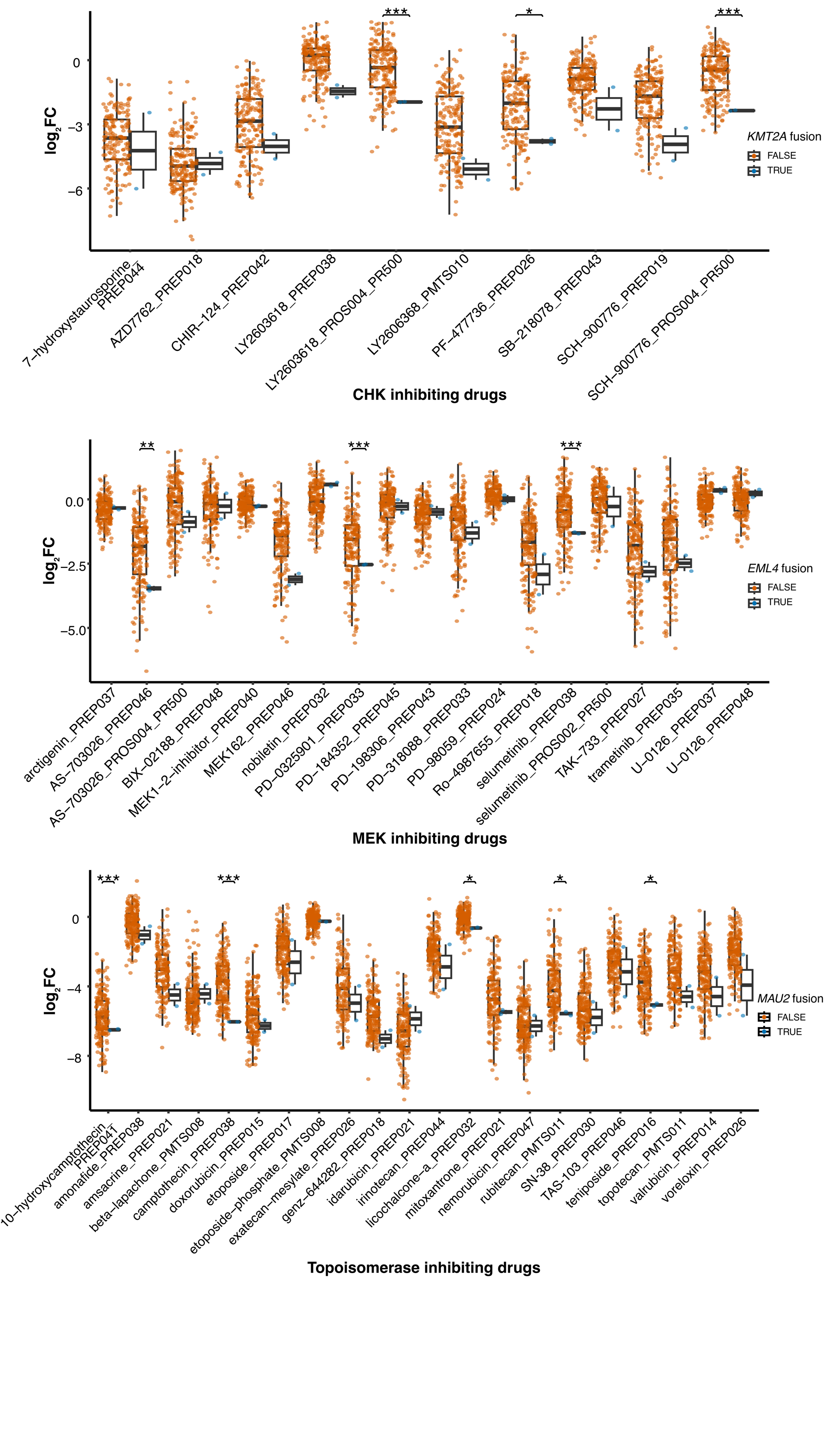

Supplement: Supplementary file 3 — Additional file 3. Additional figures from analysis of the PRISM drug repurposing dataset [file 12885_2025_15441_MOESM3_ESM.docx]
